# 

**Published:** 1903-02-21

**Authors:** 


### NOTICE TO CORRESPONDENTS.

**All MSS., Letters, Books for Review, and other matters intended for the Editor should be addressed The Editor, "The Hospital," The Hospital Building, 28 & 29 Southampton Street, Strand, W.C.**

**The Editor cannot undertake to return rejected MS., even when accompanied by stamped directed envelope.**

**Notice to Advertisers and Subscribers.**

**All Advertisements, Orders for Copies of The Hospital, and Business Communications should be addressed to THE MANAGER, (Not to the Editor), The Hospital, 28 & 29 Southampton Street, Strand, London, W.C.**

**The Cost of Subscription, post free, is as follows.—Per week, 2½d.; per quarter, 2s. 8d.; per half-year, 5s. 3d.; per annum, 10s. 6d. Foreign:—Per annum, 15s. 2d., payable, in all cases, in advance.**

**NOTICE.—Vol. XXXII. of "The Hospital" (April to September 1902) in handsome cloth binding is now on sale at the office of this Journal, price, 7s. 6d. Cases for binding the Numbers contained in this Volume 1s. 6d. Index to Vol. XXXII., 2½d., post free.**

**The Monthly Part for February is now ready. Price 6d., by post 8d.**

### THE STUDENT OF MEDICINE.

### APPOINTMENTS.

**A. F. M. Berkeley**, L.R.C.P., L.R.C.S.Edin., appointed Medical Officer to the Sixth District and Public Vaccinator for the Third District of the Bath Union. **W. J. Butcher**, M.R.C.S.Eng., L.R.C.P.Lond., D.P.H.Vict., appointed Assistant Resident Medical Officer of the Salford Union. **Alfred Campbell**, M.R.C.S., appointed Visiting Surgeon to the Gaol at Young, New South Wales. **J. Corlis**, M.D., Ch.M.McGill, appointed Health Officer for Menzies, West Australia. **R. P. Marshall**, M.R.C.S.Eng., L.R.C.P.Lond., appointed Medical Officer of one of the Districts of the St. Olave's Union. **L. J. Miskin**, M.R.C.S.Eng., L.R.C.P.Lond., appointed District Medical Officer at Koolkynie, West Australia. **J. P. Mitton**, M.R.C.S.Eng., L.R.C.P.Lond., appointed Medical Superintendent to the Devon and Cornwall Sanatorium for Pulmonary Tuberculosis, Didworthy, S. Brent, Devon. **A. G. Naylor**, M.B., C.M.Edin., appointed District Medical Officer of the Southwell Union. **Frank Oliphant**, M.B., C.M.Edin., appointed Government Medical Officer, Antigua, Leeward Islands, West Indies. **E. C. Osborn**, L.R.C.P.Lond., appointed Medical Officer to the Holborn Union Schools at Mitcham. **J. King Patrick**, M.B., Ch.B.Glasg., appointed House-Surgeon (out-door) to the Glasgow Maternity Hospital. **Arthur Thomas Price**, M.B., Ch.B.Edin., appointed Assistant Medical Superintendent at the Hospital for Insane, Toowomba, Queensland. **William Edward Redman**, L.S.A., appointed a Port Medical Officer for the Port of Picton, New Zealand. **G. H. Savage**, M.D., F.R.C.P.Lond., appointed Consulting Physician in Mental Diseases to Guy's Hospital. **Edward Fitzgerald Stapleton**, M.D., B.Ch.Dub., appointed Surgeon to the Jervis Street Hospital, Dublin. **J. H. Targett**, M.S.Lond., F.R.C.S.Eng., appointed an Obstetric Surgeon to Guy's Hospital. **Arthur J. Wallace**, M.D.Edin., appointed Surgeon to the Hospital for Women, Liverpool.

**"The Hospital" Institutional Library.**

"Hospitals and Asylums of the World." 4 Vols., with a Portfolio of Plans, complete £12 12s.
"Burdett's Hospitals and Charities, 1902." 5s.
"Cottage Hospitals, General, Fever, and Convalescent." 10s. 6d.
"Hospital Expenditure: The Commissariat." 2s. 6d.
"A Legal Handbook for the Use of Hospital Authorities." 2s. 6d.
"The Cottage Hospital Case Book or Register of Patients." 10s. and 12s. 6d.
"The Furnishing and Appliances of a Cottage Hospital." 6d.
All these are published by the Scientific Press, Ltd., and may be obtained through any bookseller, or direct from the publishers, 28 and 29 Southampton Street, Strand, London, W.C.

